# Meropenem-ANT3310, a unique β-lactam-β-lactamase inhibitor combination with expanded antibacterial spectrum against Gram-negative pathogens including carbapenem-resistant *Acinetobacter baumannii*

**DOI:** 10.1128/aac.01120-23

**Published:** 2024-01-30

**Authors:** Magdalena Zalacain, Pauline Achard, Agustina Llanos, Ian Morrissey, Stephen Hawser, Kirsty Holden, Eleanor Toomey, David Davies, Simon Leiris, Carole Sable, Adeline Ledoux, Justine Bousquet, Jérôme Castandet, Clarisse Lozano, Martin Everett, Marc Lemonnier

**Affiliations:** 1 Antabio SAS, Labège, France; 2 IHMA Europe, Monthey/VS, Switzerland; 3 Evotec, Macclesfield, United Kingdom; Shionogi Inc., Chūō-ku, Osaka, Japan

**Keywords:** meropenem, ANT3310, β-lactamase inhibitor, *A. baumannii*, carbapenem-resistance

## Abstract

ANT3310 is a novel broad-spectrum diazabicyclooctane serine β-lactamase inhibitor being developed in combination with meropenem (MEM) for the treatment of serious infections in hospitalized patients where carbapenem-resistant Gram-negative pathogens are expected. In this study, we evaluated the *in vitro* antibacterial activity of MEM in the presence of ANT3310 at 8 µg/mL against global clinical isolates that included *Acinetobacter baumannii* (*n* = 905), carbapenem-resistant Enterobacterales (CRE), carrying either oxacillinase (OXA) (*n* = 252) or *Klebsiella pneumoniae* carbapenemase (KPC) (*n* = 180) carbapenemases, and *Pseudomonas aeruginosa* (*n* = 502). MEM was poorly active against *A. baumannii,* as were MEM-vaborbactam, ceftazidime-avibactam, aztreonam-avibactam, cefepime-taniborbactam, cefepime-zidebactam, and imipenem-relebactam (MIC_90_ values of ≥32 µg/mL). On the other hand, MEM-ANT3310 displayed an MIC_90_ value of 4 µg/mL, similar to that observed with sulbactam-durlobactam, a drug developed to specifically treat *A. baumannii* infections. ANT3310 (8 µg/mL) additionally restored the activity of MEM against OXA- and KPC-producing CREs decreasing MEM MIC_90_ values from >32 µg/mL to 0.25 and 0.5 µg/mL, respectively. The combination of 8 µg/mL of both MEM and ANT3310 prevented growth of 97.5% of *A. baumannii* and 100% of OXA- and KPC-positive CREs, with ~90% of *P. aeruginosa* isolates also displaying MEM MICs ≤8 µg/mL. Furthermore, MEM-ANT3310 was efficacious in both thigh and lung murine infection models with OXA-23 *A. baumannii*. This study demonstrates the potent *in vitro* activity of the MEM-ANT3310 combination against both carbapenem-resistant *A. baumannii* and Enterobacterales clinical isolates, a key differentiator to other β-lactam/β-lactamase combinations.

## INTRODUCTION

Antimicrobial resistance (AMR) is a significant and increasing global public health threat, with an estimated 1.3 million deaths attributable to bacterial AMR globally in 2019 (1). Increasing resistance, together with limited approvals of new antibiotics, has led to an AMR crisis, as highlighted by the scientific community and recognized in the highest political spheres (2–4).

The US Center for Disease Control and the World Health Organization have identified Gram-negative pathogens as being of particular concern because they are becoming resistant to nearly all antibiotics routinely considered for treatment (5, 6). Specifically, carbapenem-resistant *Acinetobacter baumannii* (CRAB) has been classified as a Priority 1 pathogen by both organizations. CRAB was responsible for ~8,500 infections in hospitalized patients in the USA during 2017 (5). Nearly half (43.6%) of the *Acinetobacter* spp. isolates reported by 30 European Union/European Economic Area (EU/EEA) countries to the European Antimicrobial Resistance Surveillance Network (EARS-Net) for 2019 were resistant to all three antibiotic groups under surveillance (fluoroquinolones, aminoglycosides, and carbapenems) (7) and levels of multi-drug-resistant *A. baumannii* are over four times higher than for *Klebsiella pneumoniae* and *Pseudomonas aeruginosa (8*). Production of class D β-lactamases (oxacillinase; OXA) is one of the most important mechanisms of carbapenem resistance in *A. baumannii* (9).

Treatment options for carbapenem-resistant Gram-negative pathogens, and particularly CRAB, are limited and the current pharmaceutical development pipeline includes very few novel anti-infective agents which are effective against these organisms (10–12). ANT3310 is a novel broad-spectrum serine-β-lactamase (SBL) inhibitor of the diazabicyclooctane (DBO) class which strongly inhibits both *K. pneumoniae* carbapenemase (KPC) and OXA carbapenemases and potentiates the activity of meropenem (MEM) against CRAB as well as carbapenem-resistant Enterobacterales (CRE) (13).

MEM-ANT3310 is being developed for the treatment of hospital-acquired infections, such as complicated urinary tract infections (cUTI), hospital-acquired bacterial pneumonia (HABP), ventilator-associated bacterial pneumonia (VABP), and complicated intra-abdominal infections caused by Gram-negative pathogens, including those that are carbapenem-resistant, notably CRE and CRAB, and received Qualified Infectious Disease Product designation from the US Federal Drugs Administration (FDA) in January 2020 for the treatment of these serious diseases. As part of the MEM-vaborbactam clinical trials, it has already been shown that 2 g of MEM dosed every 8 h (q8h) as a 3-h IV infusion is generally safe and well tolerated (14) and achieves >90% probability of target attainment for MEM MICs ≤8 µg/mL (15, 16). This dosing regimen is being evaluated in combination with ANT3310 in Phase 1 clinical trials (ClinicalTrials.gov ID NCT05905913; EudraCT#: 2022-002258-18).

Here, we describe the inhibitory activity of ANT3310 against different OXA-type enzymes, as well as its potentiation effect on MEM activity both *in vitro*, against 905 randomly selected *A. baumannii* collected in 2018 and 2019, and *in vivo*, in thigh and lung murine infection models. In addition, this combination was also evaluated against CRE and *P. aeruginosa* isolates.

## RESULTS

### Inhibitory activity of ANT3310 and other SBL inhibitors against OXA carbapenemases

The *in vitro* activities of ANT3310 and other SBL inhibitors were evaluated against OXA carbapenemases typically present in CRE (OXA-48) and CRAB (OXA-23, -24/40, -51, and -58), with KPC-2 included as a non-OXA control (Table 1). ANT3310 showed inhibitory activity against all enzymes tested, including all the OXA enzymes from CRAB, with half-maximal inhibitory concentration (IC_50_) values ranging from 8 to 602 nM. In this respect, ANT3310 demonstrated a wider spectrum of activity than avibactam, relebactam, zidebactam, nacubactam, taniborbactam, or vaborbactam. Only durlobactam showed an equivalent spectrum, although there were noticeable differences, with durlobactam being more active against OXA-48 and ANT3310 being more active against OXA-23 and OXA-51.

**TABLE 1:** Inhibitory activity of ANT3310 and other SBL inhibitors^*b*^

	Enzyme IC_50_ (nM)					
	KPC-2	OXA-48	OXA-23	OXA-24/40	OXA-51	OXA-58
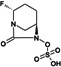 **ANT3310^*a*^**	19.5	179	32.6	602	542	8
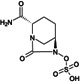 **Avi^*a*^**	7.5	252	>3,000	>10,000	>10,000	>10,000
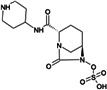 **Rel^*a*^**	17.1	>3,000	>3,000	>10,000	>10,000	>10,000
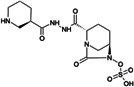 **Zid^*a*^**	38.9	>3,000	>3,000	>10,000	>10,000	>10,000
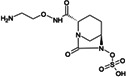 **Nac^*a*^**	27.2	>3,000	>3,000	>10,000	>10,000	>10,000
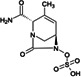 **Dur^*a*^**	5.3	3.4	346.6	208	4,303	11
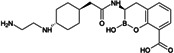 **Tan**	15.8	1,562	>3,000	>10,000	>10,000	>10,000
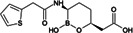 **Vab**	102	>3,000	>3,000	>1,0000	>1,0000	>10,000

^*a*^ DBO.

^*b*^ Avi, avibactam; Rel, relebactam; Zid, zidebactam; Nac, nacubactam; Dur, durlobactam; Tan, taniborbactam; Vab, vaborbactam.

### Determination of appropriate β-lactam antibiotic partner

An initial small study was performed against a set of clinical isolates including four CRAB and five SBL-positive CRE to determine the most appropriate β-lactam antibiotic partner for ANT3310. Different β-lactam antimicrobial agents, including carbapenems MEM and imipenem, and cephalosporins ceftazidime, cefepime, cefotaxime, and ceftriaxone were used in this study (data not shown).

ANT3310 potentiated the activity of all β-lactams similarly against CRE; however, against *A. baumannii* isolates, it was only effective at decreasing the MICs of the two carbapenems (data not shown). In general, addition of ANT3310 to either MEM or imipenem resulted in very similar MICs. Although both MEM and imipenem could be partnered with ANT3310 to deliver a broad-spectrum combination therapy against CRE and CRAB isolates, a decision to use MEM over imipenem as the β-lactam partner was made based on (i) the lack of requirement for cilastatin (an inhibitor of imipenem metabolism) and (ii) the fact that high doses of MEM have already been shown to be safe and tolerated in a previous combination (14).

### Lack of intrinsic activity of ANT3310

To determine whether it has intrinsic antibacterial activity, ANT3310 was tested alone against 300 OXA- or KPC-positive Enterobacterales, 502 *P*. *aeruginosa,* and 405 *A*. *baumannii* clinical isolates. No clinically significant antibacterial activity was observed for ANT3310, with MIC_50/90_ values of >64/>64 µg/mL against Enterobacterales and *P. aeruginosa* and 64/>64 µg/mL against *A. baumannii* (Table 2). Only 6 of the 1,207 isolates tested had ANT3310 MICs ≤8 µg/mL. This confirms that the role of ANT3310 in the MEM-ANT3310 combination is to potentiate MEM activity to clinically relevant levels.

**TABLE 2:** *In vitro* susceptibility data for ANT3310 against Enterobacterales, *A. baumannii,* and *P. aeruginosa*

Organism	N	Number of isolates and cumulative % inhibited at MIC (µg/mL) of:								MIC_50_	MIC_90_
			≤2	4	8	16	32	64	>64		
*A. baumannii*	405	N		1	4	1	11	38	350		
		Cum%		0.3	1.2	1.5	4.2	13.6	100	64	>64
OXA- or KPC-Enterobacterales	300	N			1		8	234	57		
		Cum%			0.7	0.7	5.3	81.0	100	>64	>64
*P. aeruginosa*	502	N				2	9	15	476		
		Cum%				0.4	2.2	5.2	100	>64	>64

### Inhibitory activity of MEM-ANT3310 and comparator agents against clinical isolates of *A. baumannii*

MEM-ANT3310 combinations and various comparator agents were tested against 905 randomly selected clinical *A. baumannii* isolates from the International Health Management Associates (IHMA) global strain collection, collected from hospital settings during 2018 (*n* = 500) and 2019 (*n* = 405). These included isolates from Europe (333; 36.8%), Asia/Pacific (209; 23.1%), North America (83; 9.2%), Latin America (94; 10.4%), Africa (95; 10.5%), and Middle East (91; 10.1%), collected from respiratory tract (60.2%), urinary tract (7.4%), skin and soft tissues (9.6%), intra-abdominal (5.3%), and bloodstream (17.1%) infections. MICs were determined across a range of concentrations and the number of strains and cumulative % inhibited at each concentration calculated (Table 3). The % susceptible and resistant have been assigned using the Clinical and Laboratory Standards Institute (CLSI) (17) and the European Committee on Antimicrobial Susceptibility Testing (EUCAST) breakpoints (BP) (18) (Table 3).

**TABLE 3:** *In vitro* susceptibility data for MEM, MEM-ANT3310, and comparator antibiotics against *A. baumannii* and CRAB isolates from 2018 and 2019 global collections

Antimicrobial agent			Number of isolates and cumulative % inhibited at MIC (µg/mL) of:												CLSI^*a*^		EUCAST	
	N		≤0.125	0.25	0.5	1	2	4	8	16	32	>32	MIC_50_	MIC_90_	% Sus	% Res	% Sus	% Res
2018 Global collection																		
All *A. baumannii*	500																	
MEM		N	7	52	42	21	9	6	5	16	64	278						
		Cum%	1.4	11.8	20.2	24.4	26.2	27.4	28.4	31.6	44.4	100	>32	>32	26.2	72.6	26.2	71.6
MEM-ANT3310 (4 µg/mL)		N	41	53	53	70	86	59	78	18	13	29						
		Cum%	8.2	18.8	29.4	43.4	60.6	72.4	88	91.6	94.2	100	2	16	88	12	–^*e*^	–
MEM-ANT3310 (8 µg/mL)		N	60	74	67	99	80	71	38	2	1	8						
		Cum%	12	26.8	40.2	60	76	90.2	97.8	98.2	98.4	100	1	4	97.8	2.2	–	–
MEM-vaborbactam (8 µg/mL)		N	18	59	28	18	9	5	5	13	69	276						
		Cum%	3.6	15.4	21	24.6	26.4	27.4	28.4	31	44.8	100	>32	>32	–	–	–	–
Imipenem-relebactam (4 µg/mL)		N	36	89	9	2	3	4	6	26	119	206						
		Cum%	7.2	25	26.8	27.2	27.8	28.6	29.8	35	58.8	100	32	>32	27.8	71.4	27.8	72.2
Ceftazidime-avibactam (4 µg/mL)		N			1	1	10	36	63	43	97	249						
		Cum%			0.2	0.4	2.4	9.6	22.2	30.8	50.2	100	32	>32	–	–	–	–
Aztreonam-avibactam (4 µg/mL)		N				1		3	13	42	163	278						
		Cum%				0.2	0.2	0.8	3.4	11.8	44.4	100	>32	>32	–	–	–	–
Cefepime-taniborbactam (4 µg/mL)		N		1	3	12	44	38	23	54	76	249						
		Cum%		0.2	0.8	3.2	12	19.6	24.2	35	50.2	100	32	>32	–	–	–	–
Cefepime:zidebactam (1:1)		N	1	2	3	26	50	22	88	132	144	32						
		Cum%	0.2	0.6	1.2	6.4	16.4	20.8	38.4	64.8	93.6	100	16	32	–	–	–	–
Amikacin		N			2	42	96	33	12	11	13	291						
		Cum%			0.4	8.8	28	34.6	37	39.2	41.8	100	>32	>32	39.2	58.2	37	63
Cefiderocol		N	123	83	111	78	52	24	3	3	2	21						
		Cum%	24.6	41.2	63.4	79	89.4	94.2	94.8	95.4	95.8	100	0.5	4	79	10.6	–	–
Colistin		N	22	287	165	15		3	2	2	4^*c*^							
		Cum%	4.4	61.8	94.8	97.8	97.8	98.4	98.8	99.2	100		0.25	0.5	–	2.2	97.8	2.2
Eravacycline		N	99	66	83	194	55	3										
		Cum%	19.8	33	49.6	88.4	99.4	100					1	2	–	–	–	–
Tigecycline		N	6	40	41	76	162	140	31	4								
		Cum%	1.2	9.2	17.4	32.6	65	93	99.2	100			2	4	–	–	–	–
CRAB^*b*^	363																	
MEM		N							5	16	64	278						
		Cum%							1.4	5.8	23.4	100	>32	>32	0	100	0	98.6
MEM-ANT3310 (4 µg/mL)		N	1	5	23	55	83	58	78	18	13	29						
		Cum%	0.3	1.7	8	23.1	46	62	83.5	88.4	92	100	4	32	83.5	16.5	–	–
MEM-ANT3310 (8 µg/mL)		N	12	24	44	86	78	70	38	2	1	8						
		Cum%	3.3	9.9	22	45.7	67.2	86.5	97	97.5	97.8	100	2	8	97	3	–	–
MEM-vaborbactam (8 µg/mL)		N							5	13	69	276						
		Cum%							1.4	5	24	100	>32	>32	–	–	–	–
Imipenem-relebactam (4 µg/mL)		N		1	1	1		4	6	26	118	206						
		Cum%		0.3	0.6	0.8	0.8	1.9	3.6	10.7	43.3	100	>32	>32	0.8	98.1	0.8	99.2
Ceftazidime-avibactam (4 µg/mL)		N						2	9	26	84	242						
		Cum%						0.6	3	10.2	33.3	100	>32	>32	–	–	–	–
Aztreonam-avibactam (4 µg/mL)		N								9	98	256						
		Cum%								2.5	29.5	100	>32	>32	–	–	–	–
Cefepime-taniborbactam (4 µg/mL)		N						2	7	36	71	247						
		Cum%						0.6	2.5	12.4	32	100	>32	>32	–	–	–	–
Cefepime:zidebactam (1:1)		N						3	63	123	142	32						
		Cum%						0.8	18.2	52.1	91.2	100	16	32	–	–	–	–
Amikacin		N			1	9	25	20	5	10	11	282						
		Cum%			0.3	2.8	9.6	15.2	16.5	19.3	22.3	100	>32	>32	19.3	77.7	16.5	83.5
Cefiderocol		N	34	63	99	72	49	21	3	3	2	17						
		Cum%	9.4	26.7	54	73.8	87.3	93.1	93.9	94.8	95.3	100	0.5	4	73.8	12.7	–	–
Colistin		N	13	210	117	13		2	2	2	4^*c*^							
		Cum%	3.6	61.4	93.7	97.2	97.2	97.8	98.3	98.9	100		0.25	0.5	–	2.8	97.2	2.8
Eravacycline		N	5	43	73	186	53	3										
		Cum%	1.4	13.2	33.3	84.6	99.2	100					1	2	–	–	–	–
Tigecycline		N			7	48	146	131	28	3								
		Cum%			1.9	15.2	55.4	91.5	99.2	100			2	4	–	–	–	–
2019 Global collection																		
All *A. baumannii*	405																	
MEM		N	7	31	18	1	5	3	9	12	66	253						
		Cum%	1.7	9.4	13.8	14.1	15.3	16.1	18.3	21.2	37.5	100	>32	>32	15.3	83.9	15.3	81.7
MEM-ANT3310 (8 µg/mL)		N	55	47	76	76	65	58	16			12						
		Cum%	13.6	25.2	44	62.7	78.8	93.1	97	97	97	100	1	4	97	3	–	–
Sulbactam-durlobactam (4 µg/mL)		N		7^*d*^	55	154	134	31	11	1		12						
		Cum%		1.7	15.3	53.3	86.2	94.1	96.8	97	97	100	1	4	–	–	–	–
Cefiderocol		N	76	72	99	64	35	19	12	8	2	18						
		Cum%	18.8	36.5	61	76.8	85.4	90.1	93.1	95.1	95.6	100	0.5	4	76.8	14.6	–	–
Colistin		N		32	296	66	5	1		1	4^*c*^							
		Cum%		7.9	81	97.3	98.5	98.8	98.8	99	100		0.5	1	–	1.5	98.5	1.5
Levofloxacin		N		61^*d*^	1	1	10	33	96	99	104^*c*^							
		Cum%		15.1	15.3	15.6	18	26.2	49.9	74.3	100		16	>16	18	73.8	15.3	84.4
CRAB^*b*^	340																	
MEM		N							9	12	66	253						
		Cum%							2.7	6.2	25.6	100	>32	>32	0	100	0	97.3
MEM-ANT3310 (8 µg/mL)		N	14	35	68	73	64	58	16			12						
		Cum%	4.1	14.4	34.4	55.9	74.7	91.8	96.5	96.5	96.5	100	1	4	96.5	3.5	–	–
Sulbactam-durlobactam (4 µg/mL)		N		2^*d*^	22	133	128	31	11	1		12						
		Cum%		0.6	7.1	46.2	83.8	92.9	96.2	96.5	95.6	100	2	4	–	–	–	–
Cefiderocol		N	32	64	93	61	34	19	9	8	2	18						
		Cum%	9.4	28.2	55.6	73.5	83.5	89.1	91.8	94.1	94.7	100	0.5	8	73.5	16.5	–	–
Colistin		N		25	251	56	4				4^*c*^							
		Cum%		7.4	81.2	97.7	98.8	98.8	98.8	98.8	100		0.5	1	–	1.2	98.8	1.2
Levofloxacin		N		9^*d*^			9	29	92	97	104^*c*^							
		Cum%		2.7	2.7	2.7	5.3	13.8	40.9	69.4	100		16	>16	5.3	86.2	2.7	97.3

^*a*^ For comparative purposes only, percent susceptible and percent resistant for MEM-ANT3310 correspond to the percentage of isolates inhibited at ≤8 µg/mL and ≥16 µg/mL, respectively. % of isolates with MEM and MEM-ANT3310 of ≤8 µg/mL MICs have been highlighted in gray.

^*b*^ MEM^R^, MICs >4 µg/mL (CLSI BP).

^*c*^ Number of isolates and cumulative % inhibited at MIC of >16 μg/mL.

^*d*^ Number of isolates and cumulative % inhibited at MIC of ≤0.25 μg/mL.

^*e*^ –, Breakpoints not defined.

Overall, 703 (78%) of isolates were resistant to MEM (MEM^R^) (CLSI, MIC ≥8 µg/mL), and can thus be designated as CRAB, with MEM^R^ rates ranging between 74% (Asia/Pacific) and 91% (Middle East) in all regions except North America where the rate was lower (40%).

#### Potentiation of MEM activity at different ANT3310 concentrations

Initially, for the first set of 500 clinical isolates (from 2018), MEM-ANT3310 combinations were tested with ANT3310 at 4 and 8 µg/mL to determine the optimal concentration for potentiation of MEM. The data showed that while addition of ANT3310 at 4 µg/mL reduced the MEM MIC_90_ from >32 µg/mL to 16 µg/mL, increasing the concentration to 8 µg/mL resulted in an additional fourfold reduction of the MEM MIC_90_ to 4 µg/mL (Table 3). Therefore, subsequent studies and comparisons versus other agents were performed using MEM-ANT3310 (8 µg/mL).

#### Activity of MEM-ANT3310 and other comparator antibiotics

**Fig 1:**
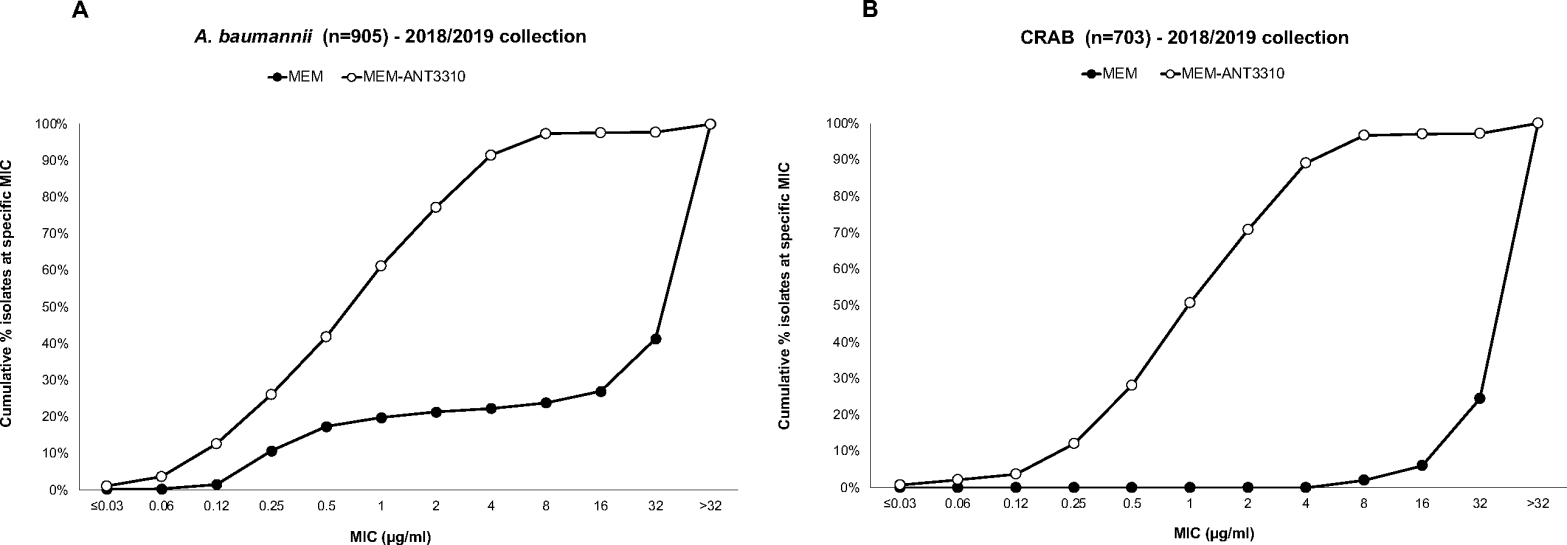
Cumulative MIC distribution of MEM-ANT3310 (8 µg/mL) against (**A**) 905 *A*. *baumannii* and (**B**) 703 CRAB clinical isolates (2018/2019 IHMA collections).

Susceptibility testing studies showed that addition of ANT3310 (8 µg/mL) reduced MEM MIC_50/90_ against the combined *A. baumannii* 2018–2019 collection (*n* = 905) from >32/>32 µg/mL to 1/4 µg/mL, with 97.5% of isolates displaying MEM-ANT3310 MICs of ≤8 µg/mL (Fig. 1A). Similarly, a reduction of MEM MIC_50/90_ from >32/>32 µg/mL to 1/8 µg/mL was observed for the subset of CRAB isolates (*n* = 703), 96.7% of which displayed MICs of ≤8 µg/mL (Fig. 1B).

**Fig 2:**
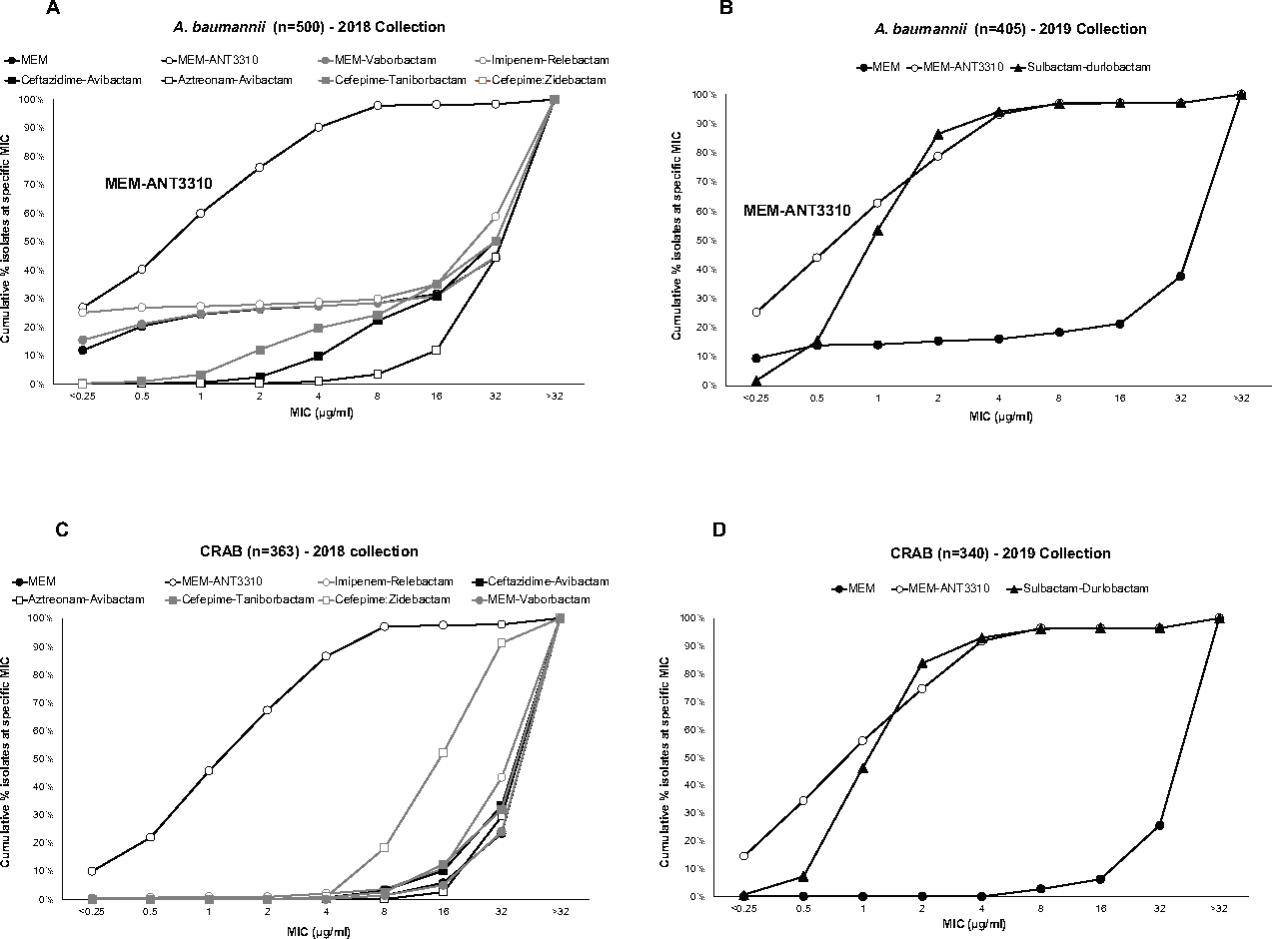
Cumulative MIC distribution of MEM-ANT3310 (8 µg/mL) and other BL/BLI combinations against *A. baumannii* or CRAB global isolates from (**A**) and (**C**) year 2018 collection (*n* = 500 and *n* = 363, respectively) and (**B**) and (**D**) year 2019 collection (*n* = 405 and *n* = 340, respectively).

Several comparator agents, either marketed or in the late stage of development, were also tested alongside MEM-ANT3310 against one or both of the 2018/2019 collections (Table 3). The marketed drugs amikacin and levofloxacin had poor activity against *A. baumannii* with MIC_90_ values ≥32 µg/mL, and only cefiderocol (MIC_90_, 4 µg/mL; 73.7% susceptible at CLSI BP), colistin (MIC_90_, 1 µg/mL; 98.1% susceptible at EUCAST BP), eravacycline (MIC_90_, 2 µg/mL; not approved for *A. baumannii*), and tigecycline (TIG) (MIC_90_, 4 µg/mL; not approved for *A. baumannii*) displayed appreciable activity (Table 3). With regard to the β-lactam/β-lactamase inhibitor (BL/BLI) combinations, neither MEM-vaborbactam, imipenem-relebactam, ceftazidime-avibactam, aztreonam-avibactam, cefepime-taniborbactam, nor cefepime-zidebactam were active against *A. baumannii,* with MIC_90_ values ≥32 µg/mL. Only MEM-ANT3310 (MIC_50/90_, 1/4 µg/mL) and sulbactam-durlobactam (MIC_50/90_, 1/4 µg/mL), a combination developed and approved solely for *Acinetobacter* infections (19), displayed significant activity (Table 3; Fig. 2A and B).

The robust activity of MEM-ANT3310 against the CRAB subset further highlighted the difference between this and other BL/BLI combinations, as only sulbactam-durlobactam had comparable activity (Table 3; Fig. 2C and D)

### Activity of MEM-ANT3310 (8 μg/mL) and comparator antibacterial agents against OXA- and KPC-CRE

**Fig 3:**
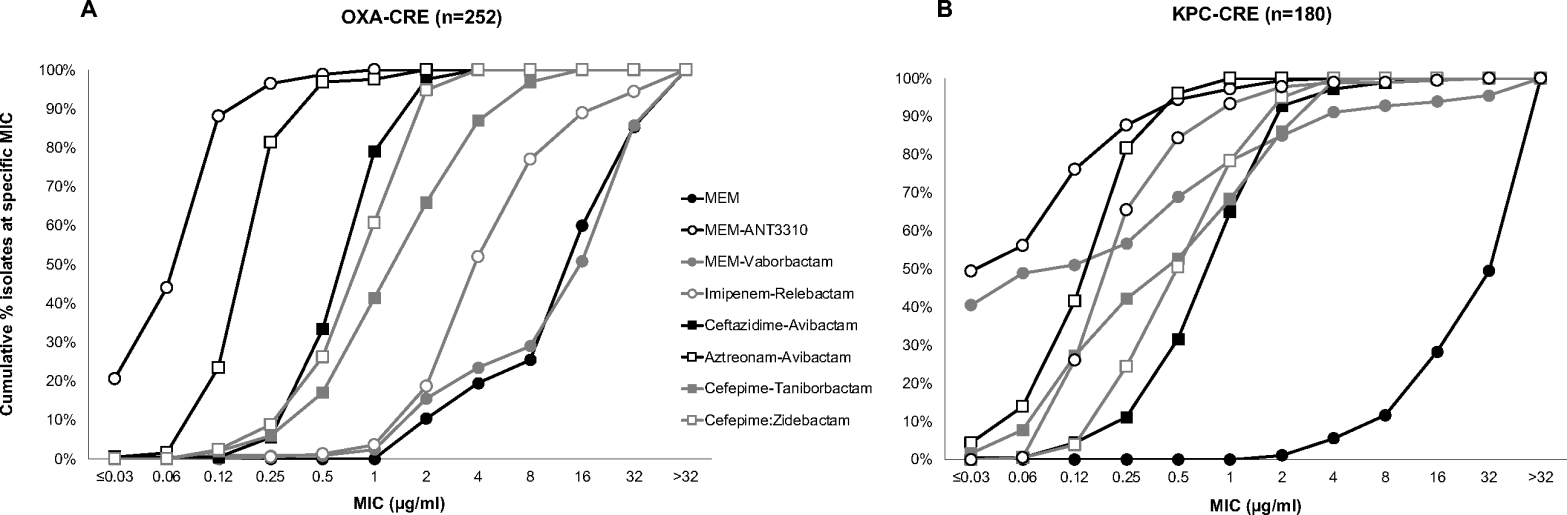
Cumulative MIC distribution of MEM-ANT3310 (8 µg/mL) and other BL/BLI combinations against (**A**) OXA-CRE (*n* = 252) and (**B**) KPC-CRE (*n* = 180) from the 2018 global collection.

The activities of MEM-ANT3310 and several comparator agents were also evaluated against 252 OXA-positive and 180 KPC-positive CRE randomly selected from the 2018 IHMA global collection of isolates. Most of the OXA-positive isolates were collected in Europe (61.1%) followed by Africa (25.0%), Asia/Pacific (7.1%), Middle East (6.3%), and Latin America (3.0%), whereas the KPC-positive CREs were mostly from Latin America (49.4%), followed by Europe (35.6%), Asia/Pacific (6.7%), North America (6.1%), and Middle East (2.2%). In both cases, the majority of isolates were from respiratory tract infections (46.8% of OXA-positive CREs and 48.3% of KPC-positive CREs). Susceptibility testing showed that addition of ANT3310 (8 µg/mL) substantially potentiated MEM antibacterial activity against these strains, with reduction of MIC_50/90_ from 16/>32 µg/mL to 0.12/0.25 µg/mL for OXA-CRE (Table 4) and 0.06/0.5 µg/mL for KPC-CRE (Table 5). In this respect, MEM-ANT3310 showed similar or superior activity against KPC- and OXA-CRE than all other BL/BLI combinations tested (Fig. 3A and B).

**TABLE 4:** Summary MIC and susceptibility data for MEM, MEM-ANT3310, and relevant comparators, against 252 OXA-producing Enterobacterales^*b*^

Antimicrobial agent(s)	MIC (µg/mL)		CLSI^*a*^		EUCAST	
	MIC_50_	MIC_90_	% Sus	% Res	% Sus	% Res
MEM	16	>32	0	89.7	10.5	74.6
MEM-ANT3310 (8 µg/mL)	0.12	0.25	100	0	–^*c*^	–
MEM-vaborbactam (8 µg/mL)	16	>32	23.4	71.0	29.0	71.0
Aztreonam-avibactam (4 µg/mL)	0.25	0.5	–	–	–	–
Cefepime-zidebactam (1:1)	1	2	–	–	–	–
Cefepime-taniborbactam (4 µg/mL)	2	8	–	–	–	–
Ceftazidime-avibactam (4 µg/mL)	1	2	100	0	100	0
Imipenem-relebactam (4 µg/mL)	4	32	3.6	81.3	18.7	81.3
Amikacin	16	>64	59.5	32.1	48.0	52.0
Cefiderocol	1	4	98.8	0.4	78.2	21.8
Colistin	0.25	8	–	23.8	76.2	23.8
Eravacycline	1	2	–	–	–	–
Tigecycline	2	4	–	–	–	–

^*a*^ For comparative purposes only, percent susceptible and percent resistant for MEM-ANT3310 correspond to the percentage of isolates inhibited at ≤8 µg/mL and ≥16 µg/mL, respectively.

^*b*^ *Citrobacter freundii* (2), *Enterobacter cloacae* (1), *Escherichia coli* (4), *Klebsiella oxytoca* (1), *K. pneumoniae* (237), *Klebsiella variicola* (1), *Klebsiella* non-speciated (1), *Providencia stuartii* (3), *Serratia marcescens* (2).

^*c*^ –, Breakpoints not defined.

**TABLE 5:** Summary MIC and susceptibility data for MEM, MEM-ANT3310, and relevant comparators, against 180 KPC-producing Enterobacterales^*b*^

Antimicrobial agent(s)	MIC (µg/mL)		CLSI^*a*^		EUCAST	
	MIC_50_	MIC_90_	% Sus	% Res	% Sus	% Res
MEM	>32	>32	0	98.9	1.1	88.3
MEM-ANT3310 (8 µg/mL)	0.06	0.5	100	0	–^*c*^	–
MEM-vaborbactam (8 µg/mL)	0.06	4	93.9	4.4	95.6	4.4
Aztreonam-avibactam (4 µg/mL)	0.25	0.5	–	–	–	–
Cefepime-zidebactam (1:1)	0.5	2	–	–	–	–
**Cefepime-taniborbactam (4 µg/mL)**	0.5	4	–	–	–	–
Ceftazidime-avibactam (4 µg/mL)	1	2	98.9	1.1	98.9	1.1
Imipenem-relebactam (4 µg/mL)	0.25	1	93.3	2.2	97.8	2.2
Amikacin	16	64	57.8	11.7	45.6	54.4
Cefiderocol	1	4	100	0	72.8	27.2
Colistin	0.25	16	–	26.7	73.3	26.7
Eravacycline	0.5	2	–	–	–	–
Tigecycline	1	4	–	–	–	–

^*a*^ For comparative purposes only, percent susceptible and percent resistant for MEM-ANT3310 correspond to the percentage of isolates inhibited at ≤8 µg/mL and ≥16 µg/mL, respectively.

^*b*^ *Enterobacter asburiae* (2), *E. cloacae* (6), *E. coli* (5), *K. oxytoca* (3), *K. pneumoniae* (159), *K. variicola* (1), *S. marcescens* (4).

^*c*^ –, Breakpoints not defined.

### Activity of MEM-ANT3310 (8 μg/mL) and comparator antibacterial agents against *P. aeruginosa* clinical isolates

**Fig 4:**
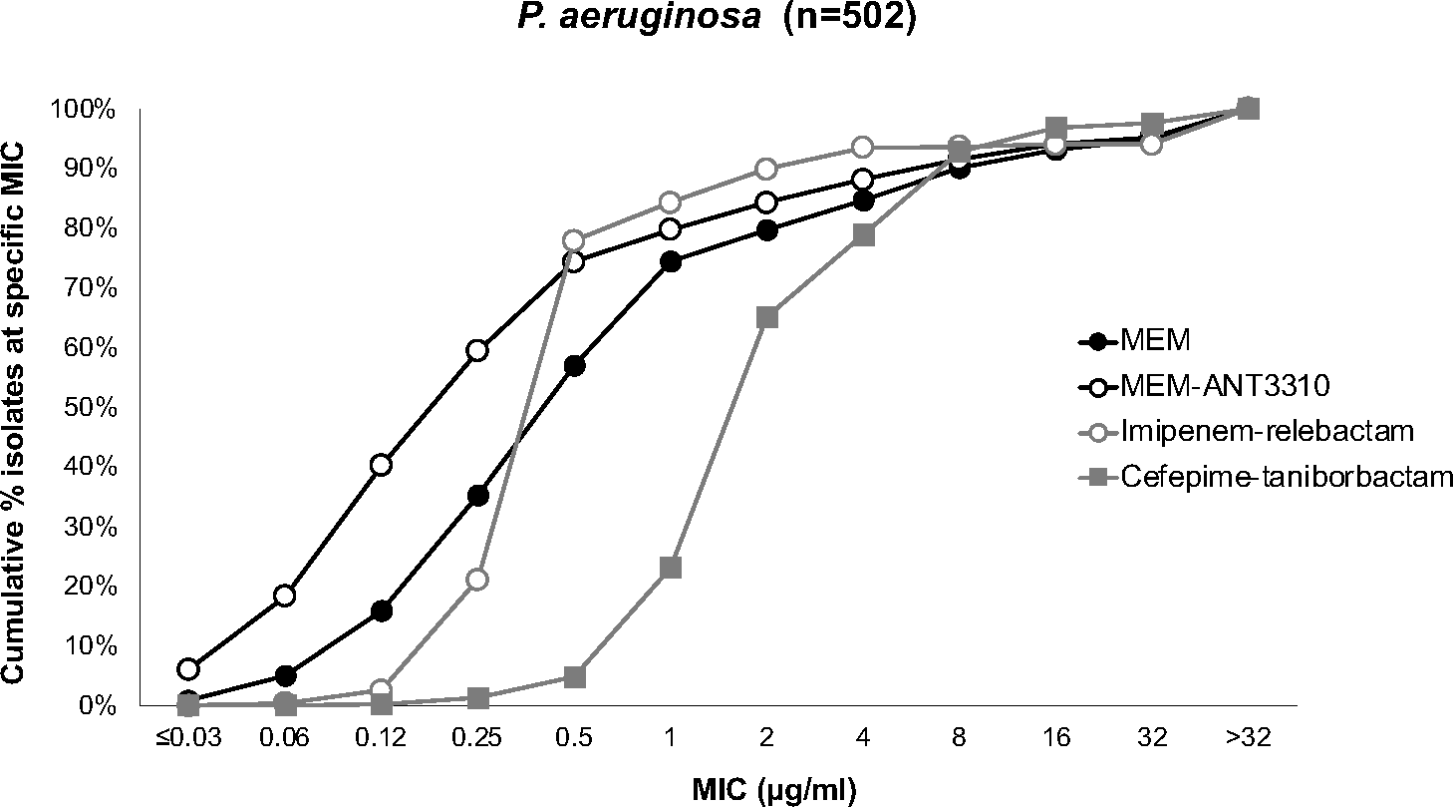
Cumulative MIC distribution of MEM, MEM-ANT3310 (8 µg/mL), and other BL/BLI combinations against 502 *P*. *aeruginosa*.

Susceptibility testing of MEM-ANT3310 and comparator antibacterial agents was also performed against 502 *P*. *aeruginosa* isolates randomly selected from the 2019 IHMA global collection. Addition of ANT3310 to MEM had a small but positive effect against strains at the lower end of the MEM MIC spectrum. This resulted in a twofold decrease in the MIC_50_ of the combination, from 0.5 to 0.25 µg/mL, although the MIC_90_ remained the same, 8 µg/mL. The activity of MEM-ANT3310 was comparable to that of imipenem-relebactam and cefepime-taniborbactam (Table 6; Fig. 4), but cefiderocol and colistin were more potent, with MIC_90_ values of 1 µg/mL.

**TABLE 6:** *In vitro* susceptibility data for MEM, MEM-ANT3310, and comparator antibiotics against 502 *P*. *aeruginosa^a^*

Antimicrobial agent	Number of isolates and cumulative % inhibited at MIC (µg/mL) of:											MIC_50_	MIC_90_
		≤0.125	0.25	0.5	1	2	4	8	16	32	>32		
Meropenem	N	80	97	109	87	27	25	27	15	9	26		
	*Cum%*	*15.9*	*35.3*	*57.0*	*74.3*	*79.7*	*84.7*	*90.0*	*93.0*	*94.8*	*100*	0.5	8
Meropenem-ANT3310 (8 µg/mL)	N	202	96	75	27	23	19	17	13	6	24		
	*Cum%*	*40.2*	*59.4*	*74.3*	*79.7*	*84.3*	*88.0*	*91.4*	*94.0*	*95.2*	*100*	0.25	8
Imipenem-relebactam (4 µg/mL)	N	13	93	285	32	28	18	1	2		30		
	*Cum%*	*2.6*	*21.1*	*77.9*	*84.3*	*89.8*	*93.4*	*93.6*	*94.0*	*94.0*	*100*	0.5	4
Cefepime-taniborbactam (4 µg/mL)	N	1	6	17	92	211	69	70	20	4	12		
	*Cum%*	0.2	*1.4*	*4.8*	*23.1*	*65.1*	*78.9*	*92.8*	*96.8*	*97.6*	*100*	2	8
Cefiderocol	N	227	125	92	33	16	7	1		1			
	*Cum%*	*45.2*	*70.1*	*88.4*	*95.0*	*98.2*	*99.6*	*99.8*	*99.8*	*100*		0.25	1
Colistin	N	1	4	21	452	21	2		1				
	*Cum%*	*0.2*	*1.0*	*5.2*	*95.2*	*99.4*	*99.8*	*99.8*	*100*			1	1

^*a*^ % of isolates at MIC_50_ and MIC_90_ values have been highlighted in light and dark gray, respectively.

### *In vivo* efficacy of MEM and MEM-ANT3310 against OXA-23 *A. baumannii* in thigh and lung murine infection models

**Fig 5:**
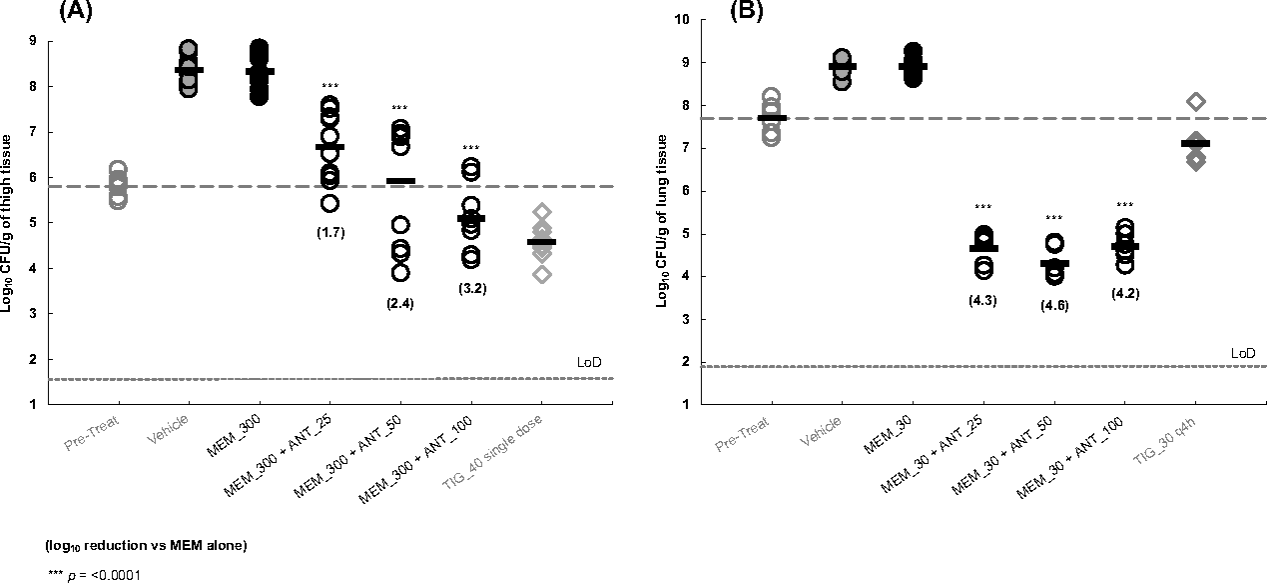
*In vivo* efficacy of MEM and MEM-ANT3310 against OXA-23 *A. baumannii* in murine (**A**) thigh and (**B**) lung infection models. MEM, MEM-ANT3310, and TIG MICs against this isolate are, respectively, 64, 4, and 2 µg/mL.

The ability of ANT3310 to restore MEM activity *in vivo* was evaluated in both murine thigh and lung infection models with *A. baumannii* ACC00445 (OXA-23). Due to its short half-life in mice, it is necessary to dose MEM frequently to maintain plasma levels above the MIC for 40% of the dosing interval. Consequently, efficacy studies in the thigh model were performed dosing MEM IV at 300 mg/kg every 2 h, as this results in a %T above 8 µg/mL equivalent to that achieved by 2 g of MEM administered q8h by 3 h IV infusion in humans (20), which is the dosing regimen proposed for the MEM-ANT3310 combination. In this 9-h model, MEM was dosed either alone or in combination with ANT3310 at 25, 50, or 100 mg/kg, with TIG included as a positive control. No reductions in bacterial counts were observed in mice treated with MEM at 300 mg/kg compared to the vehicle-treated group, but addition of ANT3310 at 25, 50, and 100 mg/kg demonstrated, respectively, a statistically significant reduction of 1.7, 2.4, and 3.2 log_10_ colony forming units (CFUs) versus MEM alone (*P* < 0.0001) (Fig. 5A). The combination of MEM-ANT3310 resulted in stasis at 300/50 mg/kg and had a killing effect at 300/100 mg/kg.

Lower doses of MEM were necessary to achieve efficacy in the 14-h respiratory tract infection model. No efficacy was observed when MEM was dosed IV at 30 mg/kg but addition of ANT3310 at 25, 50, and 100 mg/kg demonstrated a statistically significant reduction of 4.3, 4.6, and 4.2 log_10_ CFUs, respectively, versus MEM alone (*P* < 0.0001), with the combination of MEM-ANT3310 at 30/25 mg/kg reducing bacterial counts 3-log_10_ below stasis (Fig. 5B).

## DISCUSSION

In this study, we determined the inhibitory activity of the new SBL inhibitor ANT3310 against key carbapenemase enzymes and evaluated its ability to potentiate the antimicrobial activity of MEM against carbapenem-resistant clinical isolates.

ANT3310 showed strong inhibition of both KPC and OXA enzymes, including those from *A. baumannii* (OXA-23, OXA-24/40, OXA-51, OXA-58), which are largely responsible for high-level carbapenem resistance in this organism (9). Only durlobactam showed an equivalent spectrum, although it was less active against OXA-23 and OXA-51 than ANT3310.

The combination of MEM with ANT3310 was tested, alongside comparator drugs, against large numbers of *A. baumannii* (*n* = 905), OXA-CRE (*n* = 252), KPC-CRE (*n* = 180), and *P. aeruginosa* (*n* = 502), randomly selected from a large global collection. In these studies, MEM-ANT3310 demonstrated potent activity against all populations tested, with the addition of ANT3310 (8 µg/mL) decreasing the MEM MIC_90_ from >32 µg/mL to 0.5 µg/mL against KPC-CRE, from >32 µg/mL to 0.25 µg/mL against OXA-CRE, and from >32 µg/mL to 4 µg/mL versus *A. baumannii*. As 90% of *P. aeruginosa* isolates have MEM MICs ≤8 µg/mL and the MEM dosing regimen (2 g, q8h) being evaluated in combination with ANT3310 achieves >90% probability of target attainment for that MIC (15, 16), the MEM-ANT3310 combination would also provide coverage of *P. aeruginosa*.

In contrast, none of the marketed single-agent drugs tested (amikacin, eravacycline, tigecycline, colistin, and cefiderocol) provided adequate clinical coverage (>90% at approved clinical BP) against OXA- or KPC-CRE strains, and only colistin, whose utility is limited by nephrotoxicity (21), provided adequate coverage against *A. baumannii*. In fact, the 2023 Infectious Diseases Society of America (IDSA) guidelines recommend against the use of colistin to treat CRE infections (22), whereas the FDA does not recognize the CLSI BP for either CRE or *A. baumannii* and also recommends the use of alternative agents (23).

In addition, although cefiderocol has activity against CRE and *A. baumannii*, including CRAB, and is approved in the USA for the treatment of cUTI, HABP, and VABP, and in the European Medicines Agency (EMA) for the treatment of infections due to aerobic Gram-negative organisms, the recent publication of lower susceptibility BPs than originally proposed for Enterobacterales [EUCAST susceptible BP of ≤2 µg/mL (18) instead of CLSI BP of ≤4 µg/mL] and for *A. baumannii* [FDA susceptible BP of ≤1 µg/mL (24) instead of CLSI BP of ≤4 µg/mL] limits its clinical utility.

Of the BL/BLI combinations tested, all showed similarly strong inhibitory activity against KPC-CRE, whereas only MEM-ANT3310, ceftazidime-avibactam, and cefepime-zidebactam demonstrated potent activity against OXA-CRE. Furthermore, only MEM-ANT3310 and sulbactam-durlobactam, which was recently approved by the FDA for the treatment of HABP and VABP due to *Acinetobacter* ABC complex (under the trade name Xacduro), showed potent activity against *A. baumannii* isolates. Therefore, no antibacterial agent, either marketed or in late clinical development, possesses clinical coverage of both CRE and CRAB isolates and MEM-ANT3310 was the only agent tested that showed potent activity against both these important pathogen classes.

We have previously reported the *in vivo* efficacy of MEM-ANT3310 against CRE strains in a murine thigh infection model (13); here, we have demonstrated that MEM-ANT3310, delivered IV, is equally efficacious against an OXA-23 CRAB isolate in a similar mouse thigh infection model at equivalent doses (25, 50, and 100 mg/kg). Furthermore, we have demonstrated that ANT3310 shows effective potentiation of MEM against the same CRAB strain in a mouse lung infection, and at even lower doses than for the thigh infection model, indicating good distribution and retention of activity of ANT3310 in lung tissues, and supporting the potential for MEM-ANT3310 as an effective combination to treat respiratory infections.

In summary, MEM-ANT3310 has excellent *in vitro* and *in vivo* activities against key carbapenem-resistant Gram-negative pathogens, including KPC-CRE, OXA-CRE, and CRAB, and shows broad *in vitro* coverage of *P. aeruginosa*. MEM-ANT3310 is thus clearly differentiated from other BL/BLI combinations, all of which display limited activity against one or more key pathogen groups, and shows strong potential for development as a best-in-class treatment for serious Gram-negative nosocomial infections.

## MATERIALS AND METHODS

### Compounds

The following antimicrobial agents (doubling dilution range) were tested in susceptibility studies: MEM (0.004 to 32 µg/mL), ANT3310 (2 to 64 µg/mL), MEM-ANT3310 (4 µg/mL) (0.03/4 to 32/4 µg/mL), MEM-ANT3310 (8 µg/mL) (0.03/8 to 32/8 µg/mL), MEM-vaborbactam (0.004/8 to 32/8 µg/mL), ceftazidime-avibactam (0.015/4 to 32/4 µg/mL), aztreonam-avibactam (0.015/4 to 32/4 µg/mL), imipenem-relebactam (0.015/4 to 32/4 µg/mL), cefepime-taniborbactam (0.015/4 to 32/4 µg/mL), cefepime-zidebactam (1:1) (0.008 to 32 µg/mL), cefiderocol (0.03 to 32 µg/mL), amikacin (0.12 to 64 µg/mL), colistin (0.12 to 16 µg/mL), eravacycline (0.015 to 32 µg/mL), tigecycline (0.015 to 32 µg/mL), levofloxacin (0.25 to 16 µg/mL), and sulbactam-durlobactam (0.25/4 to 64/4 µg/mL).

ANT3310, relebactam, vaborbactam, zidebactam, and taniborbactam were synthesized by Aragen Life Science Limited (Hyderabad, India). Aztreonam, cefepime, and levofloxacin were purchased from Sigma (Taufkirchen, Germany). Avibactam and durlobactam were obtained from Biochempartner (Wuhan, China) and Mason-Chem (Palo Alto, CA, USA), respectively. Cefiderocol was purchased from Chem Scene (Monmouth Junction, NJ, USA) or MedChemExpress (Stockholm, Sweden). Eravacycline and tigecycline were obtained from MedChemExpress (Stockholm, Sweden) and Selleckchem (Zürich, Switzerland), respectively. MEM, imipenem, ceftazidime, sulbactam, amikacin, and colistin were purchased from the U.S. Pharmacopeia (Rockville, MD, USA).

### Bacterial isolates

All isolates were randomly selected from the frozen culture collection of IHMA collected in 2018 and 2019. Matrix-assisted laser desorption ionization-time of flight mass spectrometry was used to confirm the identity of the organisms tested (Bruker Daltonics, Bremen, Germany).

### Antimicrobial susceptibility testing

MICs for all antimicrobial agents were determined at IHMA by broth microdilution in accordance with both CLSI (17) and EUCAST (18) guidelines. Microtiter panels were prepared by IHMA and stored at −70°C in cation-adjusted Mueller Hinton broth (CAMHB) (Becton Dickinson). Iron-depleted CAMHB was used to test cefiderocol. Panels containing ANT3310 were prepared fresh on the day of testing. The panels were incubated at 35°C for 16 to 20 h in ambient air before MIC endpoints were read visually. MIC values corresponded to the first well with no visible growth. Quality control testing was performed on each day of testing with *E. coli* ATCC 25922, *P. aeruginosa* ATCC 27853, *K. pneumoniae* ATCC 700603 and BAA 1705, and *A. baumannii* NCTC 13304. MICs were interpreted using 2023 CLSI (17) and EUCAST (18) BPs.

### *In vitro* SBL inhibition assay and IC_50_ determination

Enzyme inhibition assays were performed as previously described (25) with purified KPC-2 (10 ng/well), OXA-48 (4.7 ng/well), OXA-23 (1.65 ng/well), OXA-24 (60 ng/well), OXA-51 (125 ng/well), and OXA-58 (125 ng/well) in 10 mM HEPES buffer (pH 7.5) (supplemented with 50 mM NaHCO_3_ for OXA-23, OXA-24, OXA-51, and OXA-58 assays) in 96-well microtiter plates. Nitrocefin (Sigma-Aldrich, St Louis, MO, USA), a chromogenic cephalosporin antibiotic (λ, 482 nm; ε_M_, 15,000 M^−1^.cm^−1^), was used as a reporter substrate at 100 µM for KPC-2, OXA-23, and OXA-48, at 200 µM for OXA-24 and OXA-51, and at 50 µM for OXA-58. Enzymes were preincubated in the presence of varying concentrations of each compound (0.006 to 3000 nM, diluted twofold in dimethyl sulfoxide (DMSO) for KPC-2, OXA-23, and OXA-48 assays and 0.51 to 10,000 nM diluted threefold in DMSO for OXA-24, OXA-51, and OXA-58) for 10 min at 30°C. Nitrocefin was then added and its hydrolysis by the uninhibited enzyme fraction was followed at an absorbance wavelength of 482 nm for 10 min at 30°C using Envision UV fluorescence plate reader (Perkin Elmer, Waltham, MA, USA). Hydrolysis rate data were used to determine inhibitory activities, expressed in IC_50_ values [corresponding to the compound concentration (µM) required to inhibit 50% of enzymatic reaction].

### Animals

Specific pathogen-free male CD-1 mice (11–15 g on receipt) (Charles River Laboratories, Margate, Kent, UK) were allowed to acclimatize for ~10 days then rendered neutropenic by immunosuppression with cyclophosphamide by intraperitoneal injection at 150 mg/kg 4 days before infection and 100 mg/kg 1 day before infection. The immunosuppression regime leads to neutropenia starting 24 h post administration of the first injection continuing throughout the study. Efficacy studies were performed at Evotec, Alderley Park, Cheshire, UK.

### Murine thigh infection model

Mice (five per group) were infected with 0.05 mL of a suspension of *A. baumannii* ACC00445 (OXA-23) by intramuscular injection under temporary inhaled anaesthesia (2.5% isofluorane in 87.5% oxygen for 3–4 min) into both thighs (4.67 × 10^5^ CFU/thigh). Vehicle (phosphate-buffered saline, PBS), MEM, and MEM-ANT3310 were administered IV at 1, 3, 5, and 7 h post-infection at 10 mL/kg. Tigecycline, dosed once IV at 40 mg/kg, was used as a positive control. One group of animals was humanely euthanized using pentobarbitone overdose 1 h post-infection to provide a pre-treatment control group. All animals in the additional groups were euthanized at the end of the study, 9 h post-infection. Thigh samples were homogenized in ice-cold sterile PBS; the homogenates were quantitatively cultured onto cystine-lactose-electrolyte-deficient (CLED) agar in triplicate and incubated at 37°C for 18–24 h before colonies were counted. The data from the culture burdens were analyzed using appropriate non-parametric statistical models (Kruskal-Wallis using Conover-Inman to make all pairwise comparisons between groups) with StatsDirect software v. 3.2.7, and compared to vehicle control. For all calculations, the thighs from each animal were treated as two separate data points even though they are not completely independent samples.

### Murine lung infection model

Mice (six per group) were infected with 0.05 mL of a suspension of *A. baumannii* ACC00445 (OXA-23) (1.23 × 10^7^ CFU/mouse) by oropharyngeal administration under temporary inhaled anaesthesia (2.5% isofluorane in 87.5% oxygen for 3–4 min). Vehicle (PBS), MEM, and MEM-ANT3310 were administered IV at 2, 4, 6, 8, 10, and 12 h post-infection at 10 mL/kg. Tigecycline (30 mg/kg), dosed IV at 2 and 8 h post-infection, was used as a positive control. One group of animals was humanely euthanized using pentobarbitone overdose 2 h post-infection to provide a pre-treatment control group. All animals in the additional groups were euthanized at the end of the study, 14 h post-infection. Lung samples were homogenized in ice-cold sterile PBS; the homogenates were quantitatively cultured onto CLED agar in triplicate and incubated at 37°C for 18–24 h before colonies were counted. The data from the culture burdens were analyzed using appropriate non-parametric statistical models (Kruskal-Wallis using Conover-Inman to make all pairwise comparisons between groups) with StatsDirect software v. 3.2.7, and compared to vehicle control.
